# Perineal Reconstruction with a Diamond-shaped Perforator Flap: A Case Report

**DOI:** 10.1097/GOX.0000000000001697

**Published:** 2018-03-13

**Authors:** Parthena I. Deskoulidi, Pantelis K. Diamantopoulos, Nikolaos A. Maltzaris, Maria D. Kotrotsiou, Konstantinos M. Benetatos, Spiros D. Stavrianos

**Affiliations:** From the * Department of Plastic Surgery, St. Savvas Cancer Hospital of Athens, Athens, Greece; † Department of Plastic Surgery, Evangelismos General Hospital of Athens, Athens, Greece; and ‡ Department of Plastic Surgery, 401 General Military Hospital of Athens, Athens, Greece.

## Abstract

Reconstruction of perineal defects is a challenging procedure. Various surgical techniques have been proposed. Compared with traditional myocutaneous flaps, pedicled perforator flaps are believed to be a less invasive option for perineal reconstruction, with better functional and cosmetic results. We present the case of a 47-year-old woman with a perianal Paget’s disease who underwent surgical excision of the lesion. The reconstructive technique was a pedicled flap based on an internal pudendal skin perforator artery. The flap was designed in a diamond-shaped pattern. Six months after the operation, the patient is disease-free with successful aesthetic and functional results. A polygonal diamond-shaped flap is an easy and reliable choice for perineal reconstruction, offering better adjustment in the perianal region and avoidance of the curvilinear perianal incision (which often leads to anal stenosis).

Perianal extramammary Paget’s disease affects individuals between the ages of 50 and 80 years and is more common in women and white skin races. Extramammary perianal Paget’s disease (EMPD) is a rare, slow-growing intraepithelial adenocarcinoma that mainly involves the vulvar, perineal, perianal, scrotal, and penile skin.^1–3^ Skin areas rich in apocrine glands, such as the genital region, are typical sites of EMPD. Signs and symptoms are skin lesions, often mistaken as eczema, that may be itchy or painful. The treatment of Paget’s disease is essentially surgical. After the wide local excision of the lesion with a safe margin, the next step is the reconstruction of the defect. This is often challenging. It is important to restore the shape, function, and volume. Skin grafting is vulnerable to trauma and infection. Given the size of the defect, some local flaps have insufficient volume or are difficult to fit into place. Pedicled perforator flaps are a better option for perineal reconstruction than myocutaneous flaps, as they avoid the use of a large designated vessel and undesirable functional and cosmetic results of the donor site area.^4,5^ Defects of posterior and lower regions of perineum can be successfully reconstructed by pedicled perforator flaps, based on internal pudental artery.^6^ Other options include perforators originating from the perineal or obturator artery. A polygonal shape of the flap can be used to avoid anal stenosis and multiple other complications.

## CASE REPORT

**Fig. 1.**
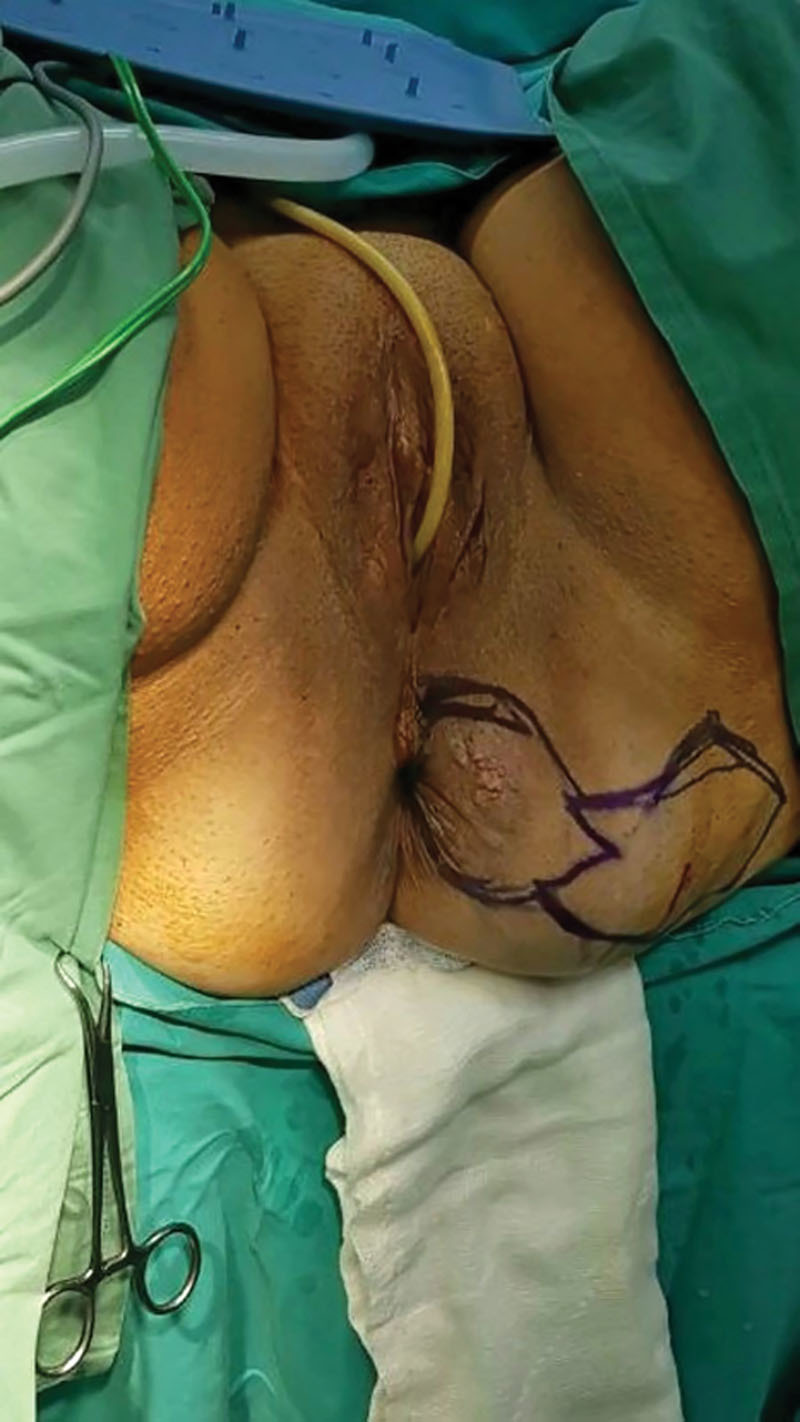
The lesion and the design of the flap in a diamond-shaped pattern.

**Fig. 2.**
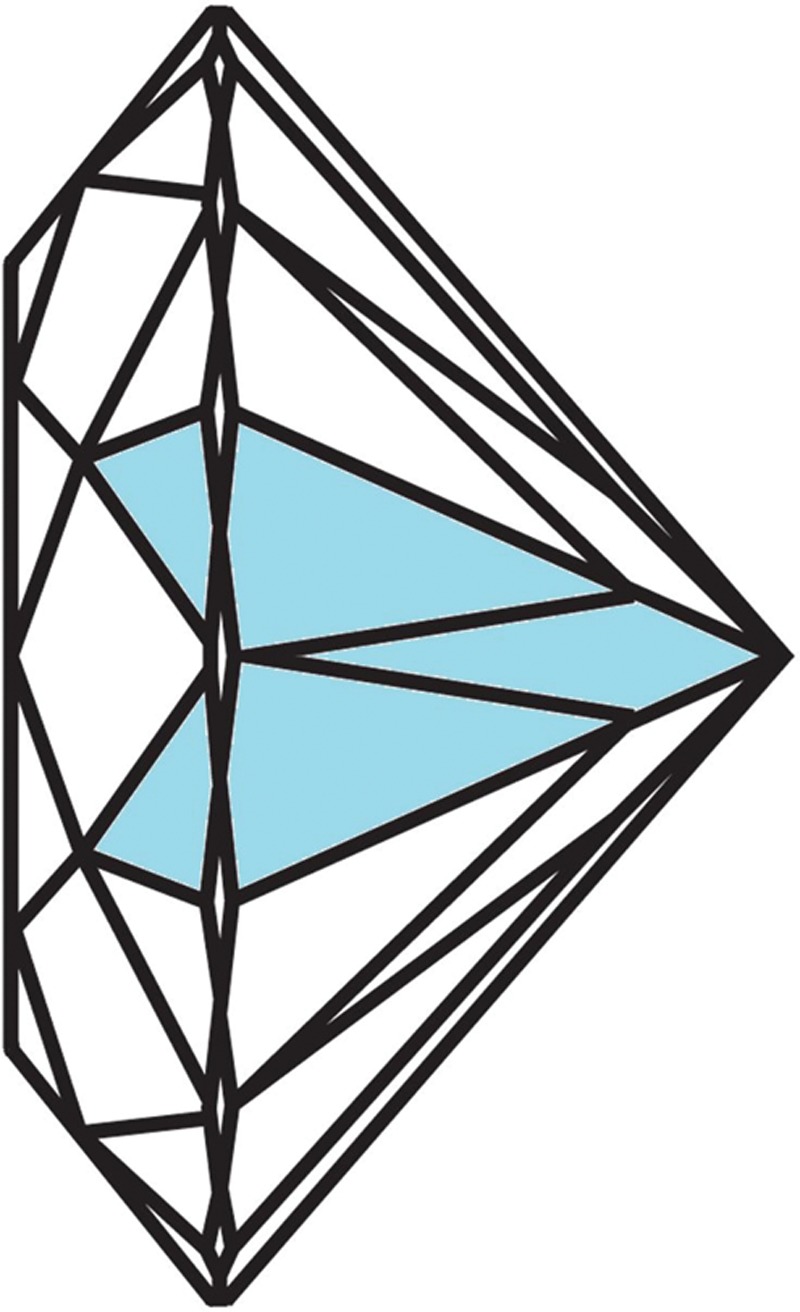
The pattern of the polygonal diamond shape.

**Fig. 3.**
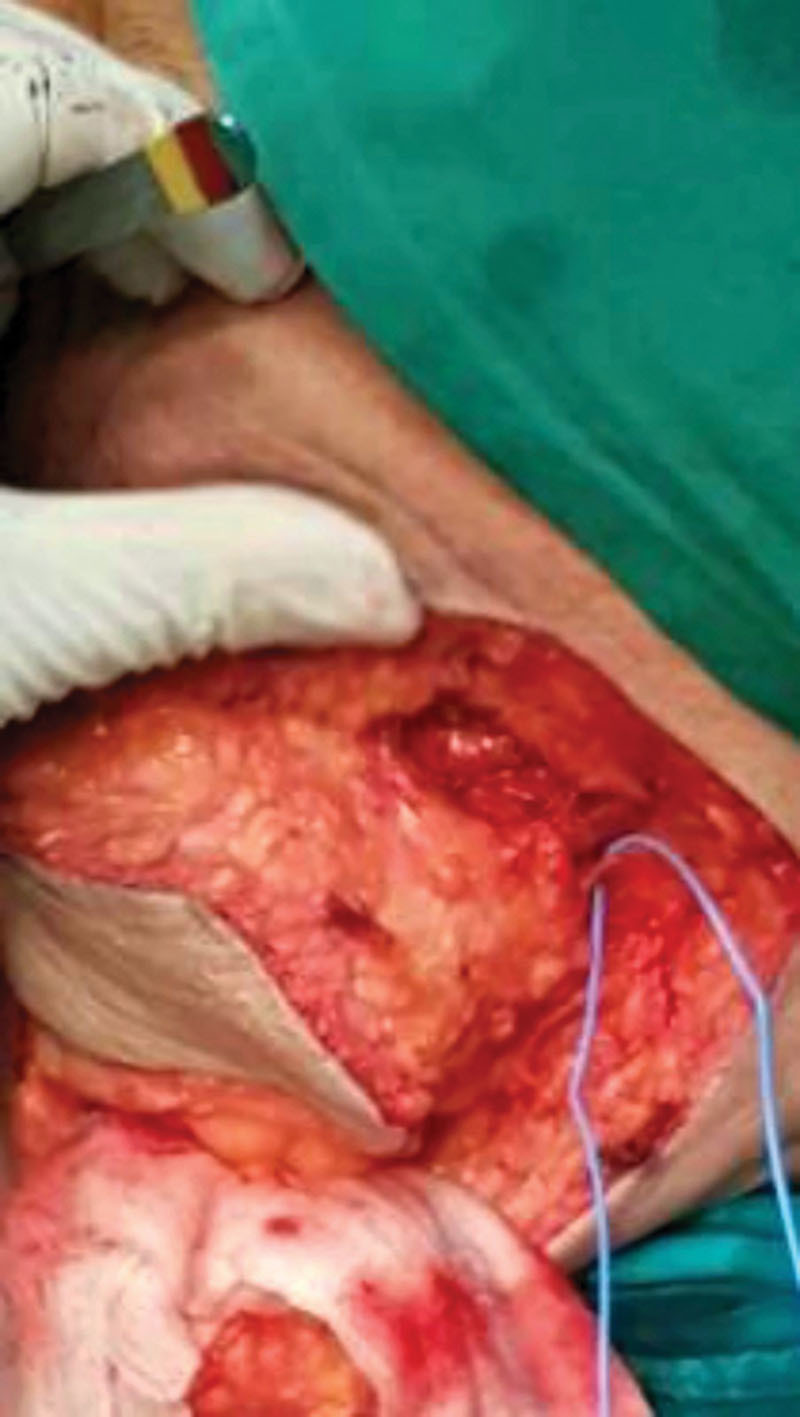
The perforator of internal pudendal artery.

**Fig. 4.**
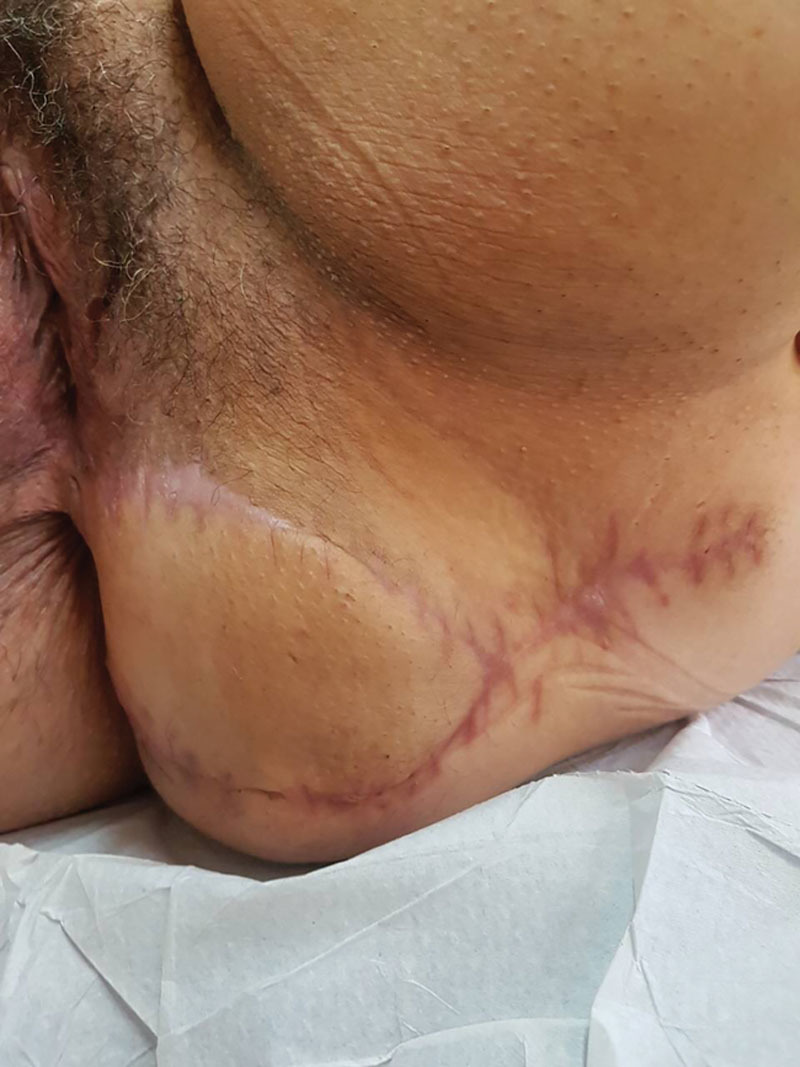
Postoperative result 4 months after surgery.

A 47-year-old woman was referred to our hospital, with a 4-year history of anal itchiness and an erythematous eczematoid skin rash in the perianal region. The woman complained that the lesion had gradually increased in size and she suffered from severe itching. A perianal shave biopsy revealed an EMPD. The preoperative testing included a complete blood count, colonoscopy, abdominal and chest computed tomography, digital mammography, an abdominal and pelvic ultrasound, and pelvic examination. The test results were negative for cancer. The procedure included a wide local excision, resulting in a semicircular defect of 3.5 cm in diameter. The reconstructive technique involved a pedicle flap based on internal pudendal artery skin perforator, which had been identified by a handheld Doppler device preoperatively.^7^ The design of the flap was in a diamond-shaped pattern (Figs. 1, 2), a modified V-Y advancement flap.^8,9^ A typical V-Y flap would not be practical because it would lead to anal stenosis. In contrast, the diamond-shaped flap was able to cover the defect, without causing anal stenosis. The perforator was identified (Fig. 3), and the subcutaneous vessels. The flap was advanced to cover the defect and sutured in place. The diamond-shaped flap covered the defect fully, with 1 quarter of its surface used to cover the intra-anal portion of the defect. The donor side was closed directly. Six months after surgery, the patient is disease-free and has returned to her activities (work, swimming, jogging). The functional and aesthetic results are successful (Fig. 4).

## DISCUSSION

Pedicled perforator flaps based on internal pudendal artery or other adjacent arteries, such as perineal, obturator, or inferior gluteal artery, are a better option for perineal reconstruction than myocutaneous flaps of gracilis,^10^ vertical rectus abdominis myocutaneous flap,^11,12^ oblique rectus abdominis myocutaneous flap, deep inferior epigastric perforator flap, avoiding the use of a large designated vessel and undesirable functional and cosmetic results of the donor-site area.

The microsurgical dissection technique of internal pudendal perforator flaps for reconstruction of perineal defects is relatively simple. The polygonal diamond shape of the flap is an easy and highly successful method for better flap insetting in the perianal region. The diamond flap allows for the reconstruction of the skin and of the anal mucosa, thus preventing anal stenosis. Furthermore, it allows the surgeon to avoid the curvilinear perianal incision, which often leads to anal stenosis.

## SUMMARY

A modified V-Y technique, the flap in a diamond-shaped pattern for perineal reconstruction based on internal pudendal artery or other adjacent arteries, such as perineal, obturator, or inferior gluteal artery, is a safe, efficient method that provides maintenance of maximal blood supply to the flap, minimal tension to the suture lines, and allows primary closure of the donor site. The diamond perforator flap is a simple, modern surgical concept, which minimizes local complications (flap necrosis), is easy to apply to the perianal region, and is not associated with anal stenosis. This flap is a modification of V-Y flap, because the typical V-Y flap would cause anal stenosis and could not be applied beyond the sphincter.
